# The use of quantitative pupillometry in brain death determination: preliminary findings

**DOI:** 10.1007/s10072-023-07251-4

**Published:** 2023-12-12

**Authors:** Pavlina Lenga, Daniel Kühlwein, Silvia Schönenberger, Jan-Oliver Neumann, Andreas W. Unterberg, Christopher Beynon

**Affiliations:** 1 Department of Neurosurgery, Heidelberg University Hospital, Im Neuenheimer Feld 400, 69120 Heidelberg, Germany; 2 Department of Neurology, Heidelberg University Hospital, Heidelberg, Germany

**Keywords:** Brain death, Quantitative pupillometry, Pupil diameter, Pupillary light reflex, Irreversibility of brain function, Organ transplantation, Pupil

## Abstract

**Purpose:**

Quantitative pupillometry (QP) has been increasingly applied in neurocritical care as an easy-to-use and reliable technique for evaluating the pupillary light reflex (PLR). Here, we report our preliminary findings on using QP for clinical brain death (BD) determination.

**Materials:**

This retrospective study included 17 patients ≥ 18 years (mean age, 57.3 years; standard deviation, 15.8 years) with confirmed BD, as defined by German Guidelines for the determination of BD. The PLR was tested using the NPi®-200 Pupillometer (Neuroptics, Laguna Hill, USA), a handheld infrared device automatically tracking and analyzing pupil dynamics over 3 s. In addition, pupil diameter and neurological pupil index (NPi) were also evaluated.

**Results:**

Intracerebral bleeding, subarachnoid hemorrhage, and hypoxic encephalopathy were the most prevalent causes of BD. In all patients, the NPi was 0 for both eyes, indicating the cessation of mid-brain function. The mean diameter was 4.9 mm (± 1.3) for the right pupil and 5.2 mm (±1.2) for the left pupil.

**Conclusions:**

QP is a valuable tool for the BD certification process to assess the loss of PLR due to the cessation of brain stem function. Furthermore, implementing QP before the withdrawal of life-sustaining therapy in brain-injured patients may reduce the rate of missed organ donation opportunities. Further studies are warranted to substantiate the feasibility and potential of this technique in treating patients and identify suitable candidates for this technique during the BD certification process.

## Introduction

Brain death (BD) is a state of irreversible cessation of all brain functions, including the cerebrum, the cerebellum, and the brain stem. However, the cardiovascular function is artificially maintained by controlled ventilation [1]. BD diagnosis is based on the clinical examination of brainstem reflexes, such as the corneal reflex, vestibulocochlear reflex, cough and gag reflexes, oculocephalic reflexes, facial movement to noxious stimuli of the trigeminal nerve, and the apnea test. One essential brain stem reflex is the pupillary light reflex (PLR), which is absent in BD patients. Usually, PLR is tested with a penlight, and bilaterally fixed pupils with a diameter > 4 mm are typically observed. However, the findings regarding the pupil size in brain-dead patients or reactivity of pupils are in disagreement, and only two studies have investigated so far the pupil size in these patients; one study suggested a pupil diameter of less than 4 mm in more than 8.5% of adult patients [2]. Additionally, abnormal pupil reactions might also occur, complicating the BD certification [3].

Quantitative pupillometry (QP) has been increasingly used in neurocritical care as an easy-to-use, affordable, and reliable technique for evaluating pupillary function [4]. Emerging data on the deployment of QP suggest that it might help detect neurological deterioration in patients with devastating intracranial pathologies, such as severe traumatic brain injury (TBI), and in monitoring intracranial hypertension [5, 6]. Notably, Couret et al. demonstrated that the standard practice in pupillary monitoring yielded inaccurate data compared to the quantitative assessment by QP. For example, the standard practice failed to detect anisocoria in half of the cases, and the degree of agreement between the pupil size measurements collected by QP versus the subjective assessments was low.

The modalities of BD determination have been a matter of debate since the Harvard Committee introduced the model of “Irreversible Coma.” For example, the BD concept definition and clinical criteria among the health care professionals and the general public are misunderstood [7]. Since the reliability of the standard practice in pupillary monitoring has been previously shown to have several limitations, QP may play a role in the clinical BD determination.

Here, we report our preliminary findings on using QP in brain-dead patients after catastrophic brain injury. Furthermore, we discuss the potential of QP during clinical BD determinations and identify potential opportunities for solid organ transplantation.

## Methods

This retrospective study included 17 consecutive patients aged ≥ 18 with confirmed BD as defined by the German Guidelines [8]. The study was approved by the ethics committee of Heidelberg University (registration number: S-788/2021).

The baseline characteristics of patients and data on cerebral injury were retrieved from their electronic records. We retrieved the cause and type of brain injury (intracranial cause/extracranial cause), localizations of primary injuries (supratentorial/infratentorial), and confirmation test modalities. All examinations were carried out according to the guidelines by two board-certified physicians in neurosurgery or neurology. Confounding conditions, such as intoxication or sedative effects, were excluded before the clinical BD determination.

### The pupillometer, assessment of pupils, and LPR

QP was assessed with the NPi-200® Pupillometer (Neuroptics, Laguna Hill, USA). The NPi-200® is a handheld infrared device automatically tracking and analyzing pupil dynamics over 3 s. The Neurological Pupil index™ (NPi) is an algorithm proprietary to NeurOptics’ pupillometers. It aims to remove subjectivity from pupillary assessment by providing a numerical value that quantifies pupillary reactivity, ranging from 0 to 5. The NPi algorithm was developed to quantify pupillary reactivity variables, such as pupil size, latency, constriction velocity, and dilation velocity [9]. The algorithm processes several factors, including constriction and dilation velocities, latency of response, and change in pupil size [9]. To calculate the NPi, the pupillometer captures and analyzes several key elements of the pupillary light reflex: baseline pupil size (this is the size of the pupil before the light stimulus is applied), latency (this is the time it takes for the pupil to start constricting after the light stimulus is applied), constriction velocity (this is the speed at which the pupil constricts after the light stimulus is applied), percentage of constriction (this is the percentage change in pupil size from the baseline to the minimum size reached after the light stimulus is applied) and dilation velocity (this is the speed at which the pupil dilates back to its original size after the light stimulus is removed).

In terms of what the numerical values signify:

- An NPi of 3 or more is generally considered within normal ranges [10].
- An NPi between 1 and 3 is indicative of a relative afferent pupillary defect (RAPD), suggesting potential neurological compromise [4].
- An NPi of 1 or less is a critical score associated with severe neurological insult, often seen in non-reactive pupils [4].

In the context of our study, the NeuroOptics pupillometer was utilized for measuring pupillary light reflex (PLR), which was automatically quantified into the Neurological Pupil index™ (NPi) using the in-built proprietary algorithm of the device. Prior to each use, the pupillometer underwent a visual inspection to verify the absence of any visible physical damage and to ensure that the device was adequately cleaned according to the manufacturer’s guidelines [11]. No user-initiated calibration was performed prior to each measurement, as the NeuroOptics pupillometer is engineered to maintain its calibration from the factory settings [11]. NPi was assessed before the clinical BD determination. Because QP is associated with low interobserver variability, we did not perform repeated examinations [4].

It is noteworthy to mention that our protocol did not involve performing repeated examinations manually. However, the NeuroOptics pupillometer has an inherent feature that automatically repeats measurements in cases of non-responsive pupils, as prescribed by the manufacturer’s “Instructions for Use.” This automatic repetition is an in-built functionality of the device, designed to enhance the reliability of the readings and operate independently from the user’s actions. In this context, even though we did not conduct repeated examinations manually, the device may have autonomously repeated measurements in certain situations. Importantly, these automatic repetitions were part of a single examination event and should not be misconstrued as repeated examinations in the traditional sense. The obtained NPi values were interpreted as part of our comprehensive neurological evaluation, thereby providing us with an objective measure of pupillary reactivity [11].

In traditional assessments without advanced tools, anisocoria of 1 mm or more was deemed clinically significant [12] (for our study, utilizing the precision of the Neuroptics Pupillometer 200, we defined a significant anisocoria as a difference of 0.2 mm, a threshold influenced by Meeker M et al., who highlighted the enhanced sensitivity of automated pupillometers in detecting subtle changes [10]).

### Confirmation of irreversibility

After completing the clinical examination of the brain stem reflexes and the apnea test, irreversibility was confirmed according to the guidelines by repeated examinations, following a period of either 12 (intracranial cause of BD) or 72 h (extracranial cause of BD), or by ancillary testing through CT-angiography or doppler sonography.

## Results

In total, 17 patients (mean age, 57.3 years; standard deviation, 15.8 years) with confirmed BD were analyzed. Intracerebral bleeding, SAH, and hypoxic encephalopathy were the most prevalent causes of BD. The details of patient characteristics, such as cerebral injury and modalities of BD confirmation testing, are summarized in Table 1.

**Table 1:** Baseline characteristics

Patient characteristics	
Mean age (y, SD)	57.3 (15.8)
Male (*n*, %)	9 (52.9)
Female (*n*, %)	8 (47.1)
Brain injury (*n*, %)	
Intracranial cause	13 (76.5)
Supratentorial	9 (52.9)
Infratentorial	4 (23.5)
Extracranial cause	4 (23.5)
Combined (intracranial/extracranial)	0 (0)
Intracerebral hemorrhage	5 (29.4)
Aneurysmal subarachnoid hemorrhage	5 (29.4)
Traumatic brain injury	3 (17.7)
Hypoxic ischemic encephalopathy	4 (23.5)
Vital parameters (*n*, SD)	
Mean systolic blood pressure (mmHg)	128.2 (15.1)
Mean body temperature (C°)	36.6 (0.7)
Mean pCO2 prior to apnea test	38.2 (2.9)
Mean pCO2 after apnea test	65.9 (4.5)
Confirmation of irreversibility (*n*, %)	
Repeat examination	7 (41.2)
Cerebral circulatory arrest (computed tomography)	9 (52.9)
Cerebral circulatory arrest (Doppler sonography)	1 (5.9)

### Pupillometry assessments

All patients with confirmed BD lacked PLR by the conventional penlight method. The QP assessment revealed a mean diameter of 4.9 mm (± 1.3) for the right pupil and 5.2 mm (±1.2) for the left (Table 2). Two patients presented with a pupil diameter of less than 4mm in both eyes, while two patients presented with clinically detected anisocoria, of whom the pupil diameter of the right eye was less than 4 mm. The NPi was 0 for both eyes, confirming the absence of PLR.

**Table 2:** Pupillometry findings

	QP findings left pupil		QP findings right pupil		Pupil size difference
	NPi	Size (mm, SD)	NPi	Size (mm, SD)	(mm, SD)
Total	0	5.2 (1.2)	0	4.9 (1.3)	0.5 (0.8)
Intracranial cause	0	5.2 (1.2)	0	4.9 (1.4)	0.5 (0.9)
Extracranial cause	0	4.9 (0.2)	0	4.8 (0.4)	0.6 (0.1)
Supratentorial brain injury	0	5.3 (0.9)	0	4.9 (1.1)	0.6 (1.0)
Infratentorial brain injury	0	5.0 (1.5)	0	4.8 (1.6)	0.4(0.4)

Although one patient with a severe acute subdural hematoma presented with clinically dilated and fixed pupils during the first BD determination, as evaluated by the flashlight pen, the QP showed pupillary function for the right eye at rest with an NPi of 3.8, which suggested the presence of brain stem reflexes; therefore, he was excluded from the present study. The patient showed spontaneous breathing during the apnea test, and a repeated QP test after 12 h found an NPi of 0. Therefore, the BD determination was repeated, which showed the complete absence of brainstem reflexes and the presence of apnea, confirming the BD diagnosis.

## Discussion

Based on internationally accepted guidelines, BD diagnosis requires the absence of brain stem functions and spontaneous breathing. Currently, a flashlight pen is a well-established tool for PLR evaluation. Following the introduction of QP in neurocritical care as a reliable tool for examining pupil reactivity, several studies have demonstrated its potential to reduce the variability of the flashlight pen examination results. To the best of our knowledge, this study systematically investigates the feasibility and potential of QP for BD certification, and we demonstrated that all brain-dead patients showed an NPi of 0, confirming the absence of PLR.

Based on the results of the present study, BD was also confirmed in patients with a pupil diameter of less than 4 mm. This is a crucial result because a diameter of more than 4 mm is essential for diagnosing BD in several countries, such as Australia, New Zealand, and Japan [13]. Our finding underscores that the absence of PLR rather than pupil size is critical for the BD diagnosis. Shlugman and colleagues showed that in a series of 148 brain-death certifications, several patients had pupil diameters below 4 mm [14], and Khandelwal et al. described cases of suspected BD patients with a pupil diameter of 3 mm, leading to a dilemma about BD assessment [13]. After a meticulous examination, all bilateral brainstem reflexes encompassing direct and indirect pupillary responses to light were absent; thus, the BD examination was initiated. Based on these facts, one might argue that the conventional examination with the flashlight cannot produce robust evidence of a PLR absence in such cases. A potential underlying mechanism for this phenomenon may include a pre-terminal event caused by intermittent discharges of damaged/dying neurons along the efferent arc, which is attributable to the release of circulating biogenic amines and neurotransmitters [15]. In BD patients, the centripetal path of pupil dilation is damaged at the brainstem level in response to light, while the centrifugal path is preserved. Consequently, the abnormal reactivity of the pupils might be explained by the presence of the ciliospinal reflex. Therefore, the deployment of QP as a standardized part of the diagnostic algorithm for the BD certification could be useful to adequately detect pupil reactions to the flashlight, avoiding potential delays in initiating further BD.

Furthermore, the study by Olgun et al. emphasizes the role of quantitative pupillometry in the assessment of brain death, similar to the core focus of our study [16]. Specifically, their observations on differences in pupillary diameter between pediatric and adult subjects provide valuable insight that can be related to our findings. In our study, we also observed differences in pupillary responses among our sample population, which underscores the importance of individualized and context-specific interpretations of pupillometry data. Like Olgun et al., we also found quantitative pupillometry to be a reliable and objective tool in assisting with brain death examination, further strengthening the body of evidence supporting its use. However, our special focus was set solely on adults with different pathologies leading to brain death and not on the young population (infant and children); thus providing refining the merit of AP in this patient cohort. Overall, the comparison between our work and the study by Olgun et al. reinforces the value and necessity of quantitative pupillometry in the complex process of brain death determination.

Anisocoria, or unequal pupil sizes, serves as a pivotal sign in neurological examinations and, particularly, in the application of pupillometry. It can indicate a diverse range of neurological conditions, and in the realm of brain injuries, it often serves as a red flag for altered intracranial dynamics, such as changes in intracranial pressure or impending brain herniation [17]. The relationship between anisocoria and lateralizing brain lesions is noteworthy. The side with the larger pupil often corresponds to the side of a mass effect or lesion causing increased pressure [17]. This can prove crucial in diagnostic and treatment protocols, particularly when combined with other neurological signs such as reduced consciousness. In the context of pupillometry, anisocoria plays an influential role in readings, particularly when interpreting the Neurological Pupil index (NPi). Discrepancies in pupil sizes can lead to variations in NPi readings between eyes, potentially introducing elements of bias or inaccuracy if not duly considered. This underlines the importance of rigorous patient assessment and individualized interpretation of pupillometry data, taking into account physical findings such as anisocoria. Moreover, the presence of anisocoria can provide valuable insights into the pathophysiology of the observed brain injuries. It might denote underlying asymmetrical or lateralizing brain pathologies that can have implications on the prognosis and treatment course of the patient. In light of these considerations, it is vital to recognize and report anisocoria when interpreting pupillometry data. This comprehensive approach not only enhances our understanding of the patient’s neurological status but also lends added accuracy to the pupillometry readings and their subsequent interpretations. The instances of anisocoria observed in our study underscore the variable presentations that can be encountered in brain-injured patients and highlight the crucial role of careful, context-aware interpretation of pupillometry data.

Compared to conventional flashlight methods, QP offers valid and rapid results. Most importantly, pupillometry is examiner-independent, and it provides additional information regarding the pupil size, constriction, and dilation [4]. In neurocritical care, QP has been increasingly applied as an easy-to-use technique enabling the rapid neurological monitoring and predicting neurological deterioration due to the early detection of intracranial hypertension in patients with TBI [5, 9]. A recent study confirmed the benefits of QP since nursing staff applying QP were able to detect anisocoria in every case, while those using the conventional flashlight yielded normal pupil reactions in only 15/30 cases [4]. Since PLR is absent in BD patients, QP displayed an NPi of 0. Larson et al. examined the usefulness of QP in patients in the post-resuscitation period in the intensive care unit and found that those patients with confirmed BD had an absent PLR, as detected by QP, while 3/9 patients thought to lack PLR based on the clinical confirmation were later found PLR positive by QP, although to a small extent [11]. In agreement with these findings, one patient in our study showed clinically absent PLR as tested by the conventional flashlight, while rest constriction and dilation of the right pupil were observed with an NPi of 3.8 by QP, suggesting the presence of midbrain function.

Solid organ transplantation offers life-saving treatment for diseases considered terminal or associated with a significant impairment in the quality of life [17]. However, the expanding demand for solid organ donation is outpacing the current organ availability due to limited organ donations. Missing a potential donor leads to preventable deaths and hurts transplant candidates. Organ donation rates could be increased by more than 10% if missed donor opportunities were avoided [18]. In this context, the use of QP may potentially avoid missed organ donation opportunities in treating patients with futile brain injury. Implementing QP before the withdrawal of life-sustaining therapy could identify potential candidates for BD determination and organ donation.

## Limitations

The present study has important limitations. Although QP represents a valid key tool for the prognosis of critically ill patients, such as those with TBI, no systematic analysis has yet assessed its BD certification value. Furthermore, the number of enrolled patients was relatively small.

## Conclusions

QP can be a valuable and robust tool for the PLR loss assessment during the BD certification process due to the cessation of brain stem function. Furthermore, the widespread belief that the diameter of the pupils of BD patients should be at least 4 mm must be redefined since BD might occur even in patients with a smaller pupil diameter; hence, the diagnosis of BD should not be excluded. However, future larger studies are required to substantiate the feasibility and establishment of this technique for BD certification. Using QP before the withdrawal of life-sustaining therapy in patients with futile brain injury could also reduce the rate of missed potential solid organ transplantations.

## Data Availability

The datasets generated during and/or analyzed during the current study are available from the corresponding author on reasonable request.

## References

[1] A definition of irreversible coma (1968) Report of the Ad Hoc Committee of the Harvard Medical School to Examine the Definition of Brain Death. JAMA 205(6):337–340. 10.1001/jama.205.6.337 5694976

[2] Takeuchi K, Takeshita H, Takakura K, Shimazono Y, Handa H, Gotoh F, Manaka S, Shiogai T (1987). Evolution of criteria for determination of brain death in Japan. Acta Neurochir.

[3] Sołek-Pastuszka J, Zegan-Barańska M, Biernawska J, Sawicki M, Iwańczuk W, Chełstowski K, Bohatyrewicz R, Dąbrowski W, Kojder K (2021). Atypical pupil reactions in brain dead patients. Brain Sci.

[4] Couret D, Boumaza D, Grisotto C, Triglia T, Pellegrini L, Ocquidant P, Bruder NJ, Velly LJ (2016). Reliability of standard pupillometry practice in neurocritical care: an observational, double-blinded study. Crit Care.

[5] Jahns F-P, Miroz JP, Messerer M, Daniel RT, Taccone FS, Eckert P, Oddo M (2019). Quantitative pupillometry for the monitoring of intracranial hypertension in patients with severe traumatic brain injury. Crit Care.

[6] Ong KL, Auerbach JD, Lau E, Schmier J, Ochoa JA (2014). Perioperative outcomes, complications, and costs associated with lumbar spinal fusion in older patients with spinal stenosis and spondylolisthesis. Neurosurg Focus.

[7] Doig CJ, Burgess E (2003). Brain death: resolving inconsistencies in the ethical declaration of death. Can J Anaesth.

[8] Irreversibler Hirnfunktionsausfall. In: Bundesärztekammer. https://www.bundesaerztekammer.de/themen/medizin-und-ethik/wissenschaftlicher-beirat/stellungnahmen-richtlinien-jahresberichte/irreversibler-hirnfunktionsausfall. Accessed 6 June 2023

[9] Chen JW, Gombart ZJ, Rogers S, Gardiner SK, Cecil S, Bullock RM (2011). Pupillary reactivity as an early indicator of increased intracranial pressure: the introduction of the Neurological Pupil index. Surg Neurol Int.

[10] Meeker M, Du R, Bacchetti P, Privitera CM, Larson MD, Holland MC, Manley G (2005). Pupil examination: validity and clinical utility of an automated pupillometer. J Neurosci Nurs.

[11] Larson MD, Muhiudeen I (1995). Pupillometric analysis of the “absent light reflex.”. Arch Neurol.

[12] Loewenfeld IE, Newsome DA (1971). Iris mechanics. I. Influence of pupil size on dynamics of pupillary movements. Am J Ophthalmol.

[13] Khandelwal A, Mishra RK, Singh S, Singh S, Rath GP (2019). Dilated pupil as a diagnostic component of brain death—does it really matter?. J Neurosurg Anesthesiol.

[14] Shlugman D, Parulekar M, Elston JS, Farmery A (2001). Abnormal pupillary activity in a brainstem-dead patient. Br J Anaesth.

[15] Loewenfeld IE, Lowenstein O (1993). The pupil: anatomy, physiology, and clinical applications.

[16] Olgun G, Newey CR, Ardelt A (2015). Pupillometry in brain death: differences in pupillary diameter between paediatric and adult subjects. Neurol Res.

[17] Black CK, Termanini KM, Aguirre O, Hawksworth JS, Sosin M (2018). Solid organ transplantation in the 21st century. Ann Transl Med.

[18] McCallum J, Yip R, Dhanani S, Stiell I (2020) Solid organ donation from the emergency department – missed donor opportunities. CJEM 22(5):701–707. 10.1017/cem.2019.482 10.1017/cem.2019.482 32122429

